# Organic Electrochemical Transistor Immuno-Sensors for Spike Protein Early Detection

**DOI:** 10.3390/bios13070739

**Published:** 2023-07-17

**Authors:** Mario Barra, Giovanna Tomaiuolo, Valeria Rachela Villella, Speranza Esposito, Aris Liboà, Pasquale D’Angelo, Simone Luigi Marasso, Matteo Cocuzza, Valentina Bertana, Elena Camilli, Valentina Preziosi

**Affiliations:** 1 CNR-SPIN, c/o Department of Physics ‘‘Ettore Pancini’’, P.le Tecchio, 80, 80125 Napoli, Italy; 2 Department of Chemical, Materials and Production Engineering—University Federico II, P.le Tecchio 80, 80125 Napoli, Italy; g.tomaiuolo@unina.it (G.T.); valeria.villella@gmail.com (V.R.V.); speranza.esposito@gmail.com (S.E.); 3 CEINGE, Advanced Biotechnologies, 80145 Napoli, Italy; 4 IMEM-CNR, Parco Area delle Scienze 37/A, 43124 Parma, Italy; aris.liboa@unipr.it (A.L.); pasquale.dangelo@imem.cnr.it (P.D.); simone.marasso@polito.it (S.L.M.); matteo.cocuzza@polito.it (M.C.); 5 Graduate School in Science and Technologies of Materials and Department of Physics, University of Parma, Parco Area delle Scienze, 7/A, 43121 Parma, Italy; 6 ChiLab, Department of Applied Science and Technology, Politecnico di Torino, 10129 Torino, Italy; valentina.bertana@polito.it (V.B.); elena.camilli@studenti.polito.it (E.C.)

**Keywords:** organic electrochemical transistors, functionalization, spike protein, long COVID, diagnostic devices

## Abstract

The global COVID-19 pandemic has had severe consequences from the social and economic perspectives, compelling the scientific community to focus on the development of effective diagnostics that can combine a fast response and accurate sensitivity/specificity performance. Presently available commercial antigen-detecting rapid diagnostic tests (Ag-RDTs) are very fast, but still face significant criticisms, mainly related to their inability to amplify the protein signal. This translates to a limited sensitive outcome and, hence, a reduced ability to hamper the spread of SARS-CoV-2 infection. To answer the urgent need for novel platforms for the early, specific and highly sensitive detection of the virus, this paper deals with the use of organic electrochemical transistors (OECTs) as very efficient ion–electron converters and amplifiers for the detection of spike proteins and their femtomolar concentration. The electrical response of the investigated OECTs was carefully analyzed, and the changes in the parameters associated with the transconductance (i.e., the slope of the transfer curves) in the gate voltage range between 0 and 0.3 V were found to be more clearly correlated with the spike protein concentration. Moreover, the functionalization of OECT-based biosensors with anti-spike and anti-nucleocapside proteins, the major proteins involved in the disease, demonstrated the specificity of these devices, whose potentialities should also be considered in light of the recent upsurge of the so-called “long COVID” syndrome.

## 1. Introduction

The uncontrolled and devastating spread of coronavirus (COVID-19 disease) that struck the world in the last two years has led to the need to find new, rapid and effective solutions to manage and reduce its diffusion. The optimization of oro-nasopharyngeal tests for the early detection of proteins involved in SARS-CoV-2 infection has been a focus of the global scientific community [1,2]. One of the main target proteins used to identify the presence of SARS-CoV-2 is represented by the spike protein, which, located at the virus surface, plays a fundamental role in penetrating host cells and initiating infection. At this stage, however, it has been recognized that the commercially available infection detection methods still exhibit limited precision, leading to false-positive results and the out-of-control diffusion of the virus [3,4,5,6]. With the progressive evolution of the COVID-19 outbreak, moreover, it has been also observed that, even after the resolution of the main symptomatology, several patients (about 10% of the initially infected population) continue to suffer from relevant symptoms, which can affect multiple organs with different degrees of impact [7]. This syndrome has been generally termed “long COVID” and is the subject of an intense global investigation at present. In this context, it was recently demonstrated that, since SARS-CoV-2 viral reservoirs may endure in the body for several months, the spike protein is among the main biomarkers that could be monitored to analyze the evolution of “long COVID” [8]. These findings further boost interest in the development of reliable biosensing platforms with the ability to combine high specificity, an excellent sensitivity and a very fast response for the detection of spike proteins.

One of the most promising approaches to address these urgent issues is given by the emerging field of organic bioelectronics, featuring outstanding performances in terms of ionic-to-electronic signal transduction and amplification. Organic electrochemical transistors (OECTs) [9,10,11] have been widely investigated as biosensors in many applications, from the detection of biomarkers [12,13,14,15,16] to neural activity [17,18,19], as well as in more general biomedical research [20,21,22,23]. Such devices exhibit a quite simple architecture, made of three electrodes (source, drain and gate) and a conductive polymer (PEDOT:PSS in our case) active layer. The polymer channel connects the source and drain contacts and is immersed in an electrolyte where the gate electrode is also positioned. The application of a voltage V_GS_ to the gate leads to the injection of ions from the electrolyte solution into the polymeric channel, thus reversibly modifying the electronic current (I_DS_) flowing between the drain and source. A PEDOT:PSS channel changes its electronic conductivity in response to the penetration of cations; hence, the related OECTs work in depletion mode following the application of positive V_G_ voltages produces a consistent decrease in the I_DS_ current. Significantly, such devices can operate at voltages lower than 1 V, making it possible for them to interact with biological fluids in the absence of relevant hydrolysis or molecule denaturation effects. However, specific care should be taken to preserve the control of the OECT response within a complex environment, including ions, nutrients, proteins, etc. In this regard, the authors have recently investigated the OECT response in human blood, outlining the effect of the corpuscular component (i.e., red blood cells) with respect to that of the liquid matrix (i.e., plasma) [24]. For biosensing applications, the OECT’s functionalization towards a specific species is one of the key points guaranteeing a proper selective response. In the last two years, various studies have focused on the early detection of antibodies or antigens involved in COVID-19, mostly regarding the spike protein [25,26]. Some recent papers analyzed the role of another of the four structural proteins involved in the infection mechanism, the nucleocapside (NP). Such a protein plays a key role in viral assembly, replication, and the host immune response regulation, making it a subject of great interest for the scientific community [27,28].

In this work, we investigate the response of PEDOT:PSS-based OECTs, driven by antibody-functionalized gate electrodes for the detection of the SARS-CoV-2 spike protein at very low concentrations, comprising femto and nanomolar values. Our study focused on the identification of the specific features of the OECT response, as this is more suitable when establishing a direct correlation with the specific concentration of the SARS-CoV-2 spike protein. Moreover, we also demonstrate that the adopted detection approach preserves its specificity behavior when analyzed as compared to the SARS-CoV-2 NP (nucleocapside) antibody.

## 2. Materials and Methods

### 2.1. Materials and Sample Preparation

Acetone, isopropanol, 3-mercaptopropionic acid (3-MPA), 11-mercaptoundecanoic acid (11-MUA), 1-ethyl-3-(3 dimethylaminopropyl) carbodiimide (EDC), N-hydroxysulfosuccinimide sodium salt (sulfo-NHS), ethanolamine, Tween 20 and phosphate-buffered saline (PBS) solution (C = 10 mM) were all purchased from Sigma–Aldrich (Missouri, MO, USA). Bovine serum albumin (BSA) was purchased by Microgem (Pozzuoli, Italy).

Primary antibodies anti-SARS-CoV-2-spike-RBD region antibody, produced in rabbit (anti-spike protein), SARS-CoV-2 NP (nucleocapside) antibody, produced in mouse and rabbit (anti-NP protein), and S protein-antigen (spike-RBD protein), i.e., SARS-CoV-2 spike Receptor Binding Domain, were purchased by Merck (Rome, Italy). Secondary antibody donkey anti-rabbit-Alexa Fluor 488 was purchased by Life-technologies (California, CA, USA).

### 2.2. OECT Fabrication

**Figure 1:**
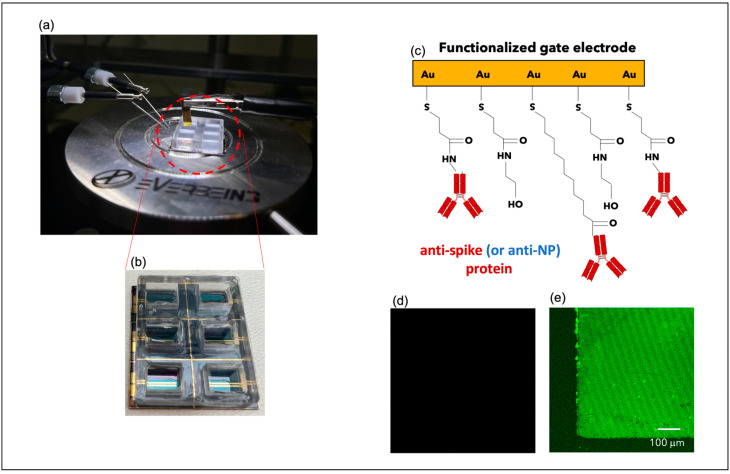
(**a**,**b**) Pictures of the layout of the employed OECT devices and of the external gate electrodes; (**c**) cartoon of the gate electrode functionalization; CLSM gate images before (**d**) and after (**e**) incubation with secondary antibodies conjugated to Alexa Fluor^®^ 488 dye.

OECTs were fabricated using a well-established protocol already described in detail elsewhere [24,29,30,31], which is briefly reviewed here. A solution of PEDOT:PSS layer (Clevios PH 1000, doped with 5 volume % ethylene glycol, 0.1 volume % dodecyl benzene sulfonic acid, and 1 wt% of GOPS (3-glycidyloxypropyl)trimethoxysilane) was spin-coated on the source and drain electrodes and subsequently patterned via photolithography (microchemicals AZ9260 resist) and etching with O_2_ plasma. A polydimethylsiloxane (PDMS) chamber, with an internal volume of 150 μL, was aligned with the PEDOT:PSS channel and irreversibly bonded on the surface (Figure 1a,b).

Electrodes were fabricated by e-beam evaporation (ULVAC EBX-14D) through the deposition of Ti/Au films (thickness 10 nm/100 nm) on Si wafer (100) finished with 1 μm of thermal oxide. The final device aspect ratio is W/L = 6 mm/200 μm = 30 (where W is the width and L is the length of the channel). The planar gate electrodes were made by a Ti/Au—10 nm/100 nm film. The latter was deposited by the evaporation systems already used for the source and drain electrodes, on a Si wafer (100) finished with 1 μm of thermal oxide (SiO_2_). Finally, they were diced in 5 × 5 or 5 × 10 mm^2^ and functionalized using the protocol described in the following.

#### 2.2.1. Gate Electrode Biofunctionalization

Gate surface was firstly cleaned in an ultrasonic bath with acetone and isopropanol for 10 min each, then the cleaned electrode was immersed in a 10 mM 3-MPA and 11-MUA (10:1 molar ratio) ethanol solution for 24 h at room temperature. This step allowed for the creation of the chemical SAM layer, exposing carboxylic groups. Then, such groups were activated by immersing the electrode in a water solution of EDC (200 mM) and NHS (50 mM) for 2 h at 25 °C. After each step, the surface was rinsed with distilled water to remove any residues and dried with air flux.

To bond the antibodies on the surface, the gate electrode was immersed in anti-spike protein solution (C = 10 nM in PBS) for 2 h at 25 °C, and then washed with 0.1% Tween PBS solution. After binding with antibodies, the gate electrode was treated with ethanolamine (1 M in PBS) and with BSA (1 μM in PBS) to saturate the remaining nonspecific sites (Figure 1c). To investigate OECT selectivity, gate electrodes were also functionalized with anti-NP protein solution at the same concentration (C = 10 nM in PBS).

The quality of the biofunctionalization protocol was tested (Section 3.1 with Figure 1 andFigure S1) by immersing gate electrodes in a solution with secondary antibodies conjugated to Alexa Fluor^®^ 488 dye (donkey anti-rabbit—Alexa Fluor 488 at dilution of 1:200, purchased by Thermofisher, Waltham, MA, USA) for 1 h at room temperature. Then, the samples were observed with confocal laser scanning microscopy (CLSM—Zeiss LSM5 Pascal) technique to assess the binding between the primary and secondary antibodies. Moreover, gate electrodes functionalized with anti-spike antibodies were analyzed by Fourier Transform Infrared Spectroscopy (FTIR) techniques. The latter was performed using Agilent Cary 630 FTIR (Agilent Technologies, Inc., Santa Clara, CA, USA), working with an ATR module in the range between 4000 and 650 nm. Before any FTIR spectrum acquisition, the samples were carefully dried on a very thin N_2_ flow for 5′ to reduce the moisture. During the analysis, more than 10 spectra were collected to find the best working conditions, i.e., environmental parameters, number of scans, resolution, overall instrument working energy and to perform several comparisons with different backgrounds.

#### 2.2.2. OECT Characterization

A Keithley two-channel multimeter (Keithley 2602B) operating by Labview code was used to characterize the electrical response of the OECT devices. Output curves were obtained by applying a voltage between gate and source, V_GS_, ranging between −0.6 and 0.6 V with a step of 0.1 V, and measuring drain-source (I_DS_) and gate-source (I_GS_) current, as a function of drain-source voltage, V_DS_ (in the range between 0 and −0.6 V). Then, curves were transferred, reporting I_DS_ by varying V_GS_ between −0.6 and 0.6 V with a step of 0.025 V at V_DS_ = −0.3 V, were recorded for all the investigated functionalized gate electrodes (i.e., with anti-spike or anti-NP proteins). In the transfer curves, each point was acquired every 2 s (Δt = 2 s). For comparison, curves at different times (Δt = 0.5 and 5 s) are reported in the Supplementary Information (Figure S3).

This protocol eas applied by using the gate electrodes functionalized with antibodies to drive the OECT, while PBS 10 mM was employed as electrolyte. Then, the functionalized gates were incubated with a spike-RBD protein from femto to nanomolar concentrations for about 20 min at room temperature and, after careful rinsing with bi-distilled water, the same electrodes were used to repeat the OECT measurement again in PBS 10 mM.

Before starting any biosensing experiment, the OECT channels were immersed in bi-distilled water for at least 2 h. Moreover, a set of preliminary transfer curves were recorded to stabilize the OECT response in PBS 10 mM. A data analysis of transfer curves could describe the OECT electrical response in terms of the current modulation values expressed as (I_DS_ − I_0_)/I_0_, where I_0_ is the baseline current, and transconductance g_m_ = δI_DS_/δV_GS_, which represents the transduction efficiency that is related to the channel current slope as a function of the voltage V_GS_. For all the experiments with bio-functionalized gate electrodes, particular care was paid to keep the immersed gating area fixed in place (A_v_ = 8 mm^2^).

The mean and standard deviation values for all transfer curves were achieved by acquiring five consecutive transfer curves and discarding the first one (generally affected by a major level of variability) to extract the various statistical parameters.

## 3. Results

### 3.1. Validation of the Gate Electrode Biofunctionalization Process

A validation step of the biofunctionalization process was initially carried out through immunofluorescence to demonstrate the presence of antibodies on the gate surface. In particular, gate electrodes previously incubated with primary antibodies were immersed in solutions with secondary antibodies conjugated to Alexa Fluor^®^ 488 dye. Since such molecules have a specific affinity to the primary antibodies, they can unequivocally demonstrate their presence on the gate surface (Figure 1d,e). Before the testing step, the electrodes were rinsed with PBS to remove any impurities and dried with cleaned air. Hence, confocal laser scanning microscopy (CLSM) images recorded before (Figure 1d) and after (Figure 1e) the functionalization step confirmed the effectiveness of the adopted protocol. A further analysis of the gate functionalization was conducted by the Fourier Transform Infrared Spectroscopy (FTIR) technique. In particular, FTIR spectra were obtained from surfaces before and after the antibody functionalization process. As shown in Figure S1, Sample A was taken from a bare cleaned Au layer; meanwhile, Sample B was recorded for a gold surface once the functionalization process with an anti-spike protein was completed. The spectrum obtained on Sample A is fully consistent with a bare gold surface, as no relevant peak was observed in the considered range. Conversely, Sample B exhibited many different peaks all across the spectrum, as the antibody itself is a protein rich in functional groups that are visible in the IR range. In this context, FTIR spectroscopy can provide important information regarding proteins’ secondary structure [32]. According to the literature, increasing the size of the molecules induces a minor sensitivity, but important information about the presence of the antibody and the success of the process can still be achieved [33]. In Figure S1, two main peaks in the secondary structure are present. The former, namely, Amide A, is located at 3252 nm and indicates the N-H stretching in resonance; meanwhile, the latter, namely, Amide I, is found at 1654 nm and shows the C=O stretching vibration. These peaks provide strong evidence of the correct bonding of the antibody on the underneath layer, representing significant markers of the presence of proteins [33,34].

### 3.2. OECT Initial Characterization

**Figure 2:**
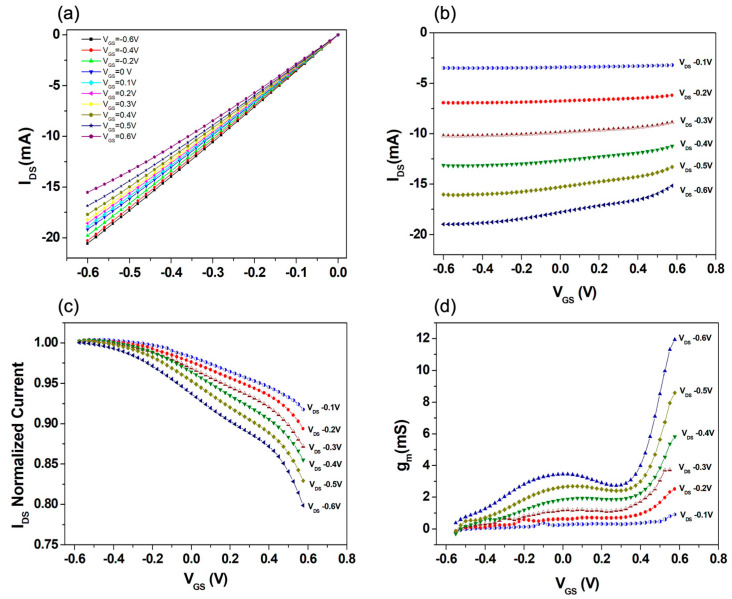
(**a**) Output curves reporting I_DS_ as a function of V_DS_ and applying V_GS_ between −0.6 and 0.6 V; (**b**) OECT transfer curves and (**c**) normalized transfer curves, with respect to the I_DS_ current at V_GS_ = −0.6 V, measured as a function of V_GS_ with V_DS_ ranging from −0.6 to −0.1 V; (**d**) transconductance g_m_ as a function of V_GS_ with V_DS_ ranging between 0.1 and −0.6 V.

Before the biosensing experiments, the OECTs investigated in this work were carefully characterized to set the optimal operating conditions. Figure 2 reports a general view of a typical response achieved with an un-functionalized (bare) gold gate electrode. As shown from the output curves in Figure 2a (with V_GS_ ranging between −0.6 V and 0.6 V, while V_DS_ was varied between 0 and −0.6 V), the devices behave correctly as depletion-mode transistors with the I_DS_ current (always in the range of mA) decreasing (in absolute value) following the application of positive V_GS_. In the output curves, only the linear and the triode regions can be observed, and this feature is to be ascribed to the gating condition that was adopted. Indeed, because of the size of the gate-immersed area (~8 mm^2^, see Materials and Methods section) and considering the volumetric capacitance (C_V_ ~ 40·F⋅cm^−3^), which can be associated with the entire electrolyte/PEDOT:PSS distributed interface, the response of the investigated OECT is mainly dictated by the electric double-layer (EDL) capacitance (C_G_) at the gate–electrolyte interface (i.e., C_G_ ≪ C_V_) [35,36]. In the framework of the model of Bernards and Malliaras [37], this condition provides a very large value (higher than 1 V) of the so-called pinch-off (V_p_) voltage, which determines the achievement of the current saturation phenomenon when V_GS_ = 0 V and V_DS_ < −|V_p_|.

Figure 2b shows a set of transfer curves recorded at different V_DS_, while, in Figure 2c, the same curves are represented as normalized with respect to the I_DS_ value measured at V_GS_ = −0.6 V. Finally, Figure 2d reports the corresponding transconductance (g_m_) values (g_m_ = δI_DS_/δV_GS_).

**Figure 3:**
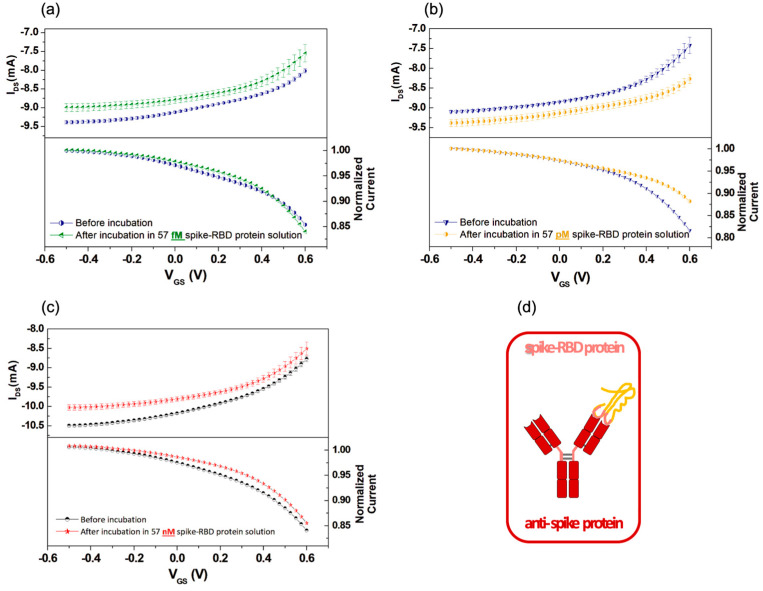
Transfer curves (top panels) and normalized transfer curves with respect to the I_DS_ current recorded at V_GS_ = −0.5 V (bottom panels) measured as a function of V_GS_ by using a functionalized gate with anti-spike protein before incubation and after incubation in solutions with (**a**) 57 femtomolar (fM) spike-RBD protein; (**b**) 57 picomolar (pM) spike-RBD protein; (**c**) 57 nanomolar (nM) spike-RBD protein; (**d**) cartoon reporting the spike antibody–antigen binding event.

These plots clearly show that by increasing the absolute value of the applied V_DS_, both the overall I_DS_ modulation and g_m_ tend to rise. In particular, the transconductance receives values in the range of mS, confirming the excellent capabilities of OECT while operating as amplifying elements [17]. It is important to outline that, at a larger |V_DS_|, the g_m_ curves exhibit the characteristic presence of a broad peak, for which the position and maximum value can be modified as a function of V_DS_. For all the transfer curves, moreover, a consistent rise in g_m_ when V_GS_ approaches 0.6 V can be observed, with this behavior becoming more and more pronounced at a larger |V_DS_|. As shown in Figure S2 of the Supplementary File, this trend is accompanied by larger values of the gate current (I_GATE_). Provided this analysis, for the biosensing experiments discussed in the following section, all the transfer curves were recorded by keeping a fixed V_DS_ = −0.3 V. This choice was motivated by the search for a good trade-off between sufficiently large values of g_m_ and I_DS_ modulation with the presence of an extended V_GS_ region (from negative values and until 0.3/0.4 V), where g_m_ assumes a mild and increasing dependence on V_GS_. As discussed in the next section, this last feature can be used to simplify the analysis of the OECT performances in terms of the detection abilities of spike-RBD protein. Significantly, under the application of V_DS_ = −0.3 V, I_GATE_ values remain constantly lower than 200 nA (Figure S2b), being four orders of magnitude smaller than the corresponding I_DS_ current throughout the analyzed V_GS_ range. Since the specific OECT response is determined by the synergistic combination of an electronic and an ionic circuit featuring typically different time dynamics as a function of the gating conditions and the active channel size [35,37], the effect of different sweep times in the transfer curve recording was also analyzed. Basically, various values of the time delay (Δt = 0.5, 2 and 5 s) between the V_GS_ application (step ΔV_GS_ = 0.02 V) and the measurement of the corresponding I_DS_ value were taken, with the goal of assessing the related impact on the transfer curves and the corresponding g_m_ values. According to the results summarized in Figure S3, if the measurements are performed too fast (namely, with Δt = 0.5 s), the devices are unable to reach a steady condition. Hence, the eventual modulation and g_m_ values (Figure 3b) are consistently reduced. In consideration of these findings, for all the measurements discussed hereafter, ∆t = 2 s was selected as being able to provide a good trade-off between the speed and accuracy of the experiments. In the absence of Faradaic reactions at the gold gating surface, the gate current (I_GATE_) plot in Figure S3c confirms the fundamental capacitive nature (i.e., displacement current) of I_GATE_ which, indeed, is strongly dependent on the overall sweep time (i.e., the faster the measurement, the larger the I_GATE_ ∝ δV_GS_/δt).

### 3.3. OECT Detection of Spike-RBD Proteins

For all the experiments devoted to the detection of SARS-CoV-2 spike **RBD** protein, before any incubation step, the OECTs were preliminarily characterized in PBS. The so-achieved transfer curves (as average of four curves consecutively measured with V_DS_ = −0.3 V; see the Materials and Methods section) were then compared with analogous data acquired after the incubation (for 20 min) in solutions containing variable concentrations of the spike-RBD protein. According to this protocol, and similar to other experimental studies [25], the gate electrode represents the sensing and disposable components of the overall device, while the PEDOT:PSS channel can be used as an ion-to-electron amplifying transducer in combination with different gating surfaces.

Following the aforementioned procedure, Figure 3a–c shows the results of three experiments at 57 fM, 57 pM and 57 nM spike-RBD protein concentrations (i.e., 1.4 pg/mL, 1.4 ng/mL, 1.4 µg/mL, respectively). Firstly, it should be mentioned that a shift in the current baseline (i.e., the almost constant I_DS_ current measured at very negative V_GS_ values) was observed in most cases, but without any clear correlation with the incubation step and the corresponding spike-RBD protein concentration. This effect is likely related to underlying ion diffusion processes, which can slightly modify the overall conductivity of the PEDOT:PSS channel over time during the different measurements.

More interestingly, as suggested by the acquired transfer curves and their normalized counterparts, shown in Figure 3 (top and bottom panels, respectively), the incubation process tends to also modify the general shape of the transfer curves. The most evident feature is the reduction in the I_DS_ slope, which is mainly observed in the intermediate V_GS_ range between slightly negative values and 0.3/0.4 V. For very negative V_GS_ values, the I_DS_ slope remains rather low, and is quite unchanged following the incubation procedure. Conversely, as previously discussed (see Section 3.2), when V_GS_ exceeds 0.3 V, the I_DS_ growth with V_GS_ becomes very steep and, in this case, turns out to be less reproducibly affected by the antibody–antigen binding occurrence. A further and clearer evidence of the I_DS_ slope decrease in the intermediate V_GS_ region, related to the incubation procedure, is given in Figure S4a–c (see the Supplementary File) directly showing the corresponding transconductance (g_m_) reduction.

**Figure 4:**
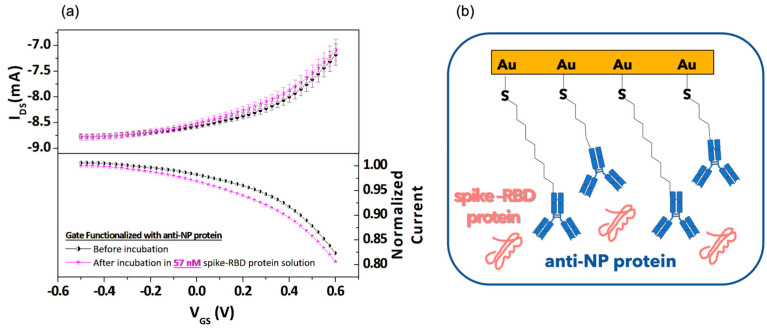
(**a**) Transfer curves (top panel) and normalized transfer curves with respect to the I_DS_ current recorded at V_GS_ = −0.5 V (bottom panel) as a function of V_GS_ by using a functionalized gate with nucleocapside (NP) antibody before and after incubation in 57 nanomolar spike-RBD protein solution; (**b**) cartoon of the anti-NP protein and spike-RBD protein unbound configuration.

For the sake of completeness, Figure 4 summarizes the results of an experiment where a gate functionalized with anti-SARS-CoV-2 NP (anti-NP) antibody was incubated in the spike-RBD protein solution with nanomolar (57 nM) concentration. At odds with the observations commented for Figure 3, here, the incubation step produced less characteristic changes and the related transconductance values were found to be slightly increased (Figure S4d) in the intermediate V_GS_ region following the incubation process. As a whole, these observations confirm that the OECT behaves coherently following the non-specific interaction of the anti-NP protein and the spike-RBD protein.

**Figure 5:**
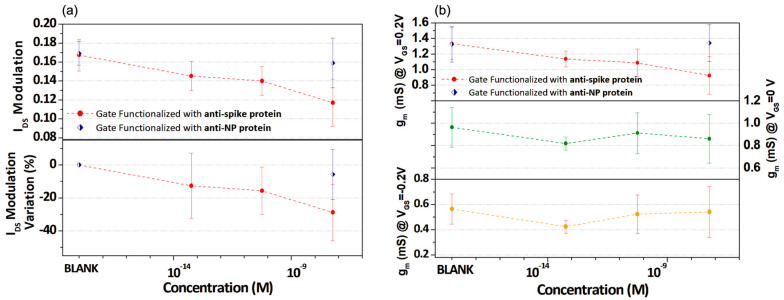
(**a**) OECT I_DS_ modulation (top panel) and its percent variation (bottom panel) as a function of spike- RBD protein concentration for a gate functionalized with spike and nucleocapside antibody. (**b**) Transconductance (g_m_) values estimated at different V_GS_ values (V_GS_ = +0.2 V top, V_GS_ = 0 V middle, V_GS_ = −0.2 V bottom) as a function of spike-RBD protein concentration for gate functionalized with spike antibody. The g_m_ values estimated at V_GS_ = 0.2 V, achieved for gate functionalized with nucleocapside antibody, are reported in the top panel of Figure 5b.

Figure 5 offers a summary of all the previously performed experiments, demonstrating the ability to identify the different concentrations of the spike-RBD protein by driving the OECTS using gate electrodes functionalized with the proper antibody. Figure 5a reports the I_DS_ modulation values given by the absolute value of (I_F_ − I_0_/I_0_), where I_F_ and I_0_ are the I_DS_ currents in the transfer curves recorded at V_GS_ = 0.6 V and V_GS_ = −0.5 V, respectively. Here, the mean values from different experiments are indicated for any spike-RBD protein concentration, with the standard deviations being the error bars. The bottom panel of Figure 5a represents the percent variation in any I_DS_ modulation value with respect to that measured prior to the corresponding incubation (BLANK). As shown, this analysis reveals the progressive reduction in the I_DS_ modulation at an increasing spike-RBD protein concentration, with the percent decrease approaching 30% for the concentrations in the nanomolar range. It is worth highlighting that the I_DS_ modulation value is largely used in the literature as a main parameter in biosensing experiments focused on the use of OECT [25,38] and that, similarly to what is observed here, percent variations ranging between a few units and tens per cent are typically reported for analyses dealing with antigen concentrations that are variable over several orders of magnitude [21,29]. In general, the I_DS_ modulation reduction can be explained by considering that the incubation process provides a further decrease in the C_G_ (i.e., the capacitance between gate and electrolyte) values, consequently attenuating the gate electrode’s overall ability to modulate the I_DS_ current.

Following the aforementioned discussion about the incubation-induced changes in the slope of the transfer curves, Figure 5b reports the transconductance values estimated at V_GS_ = +0.2 V (top panel), V_GS_ = 0 V (middle panel) and V_GS_ = −0.2 V (bottom panel) as a function of the spike-RBD protein concentration. Here, the decreasing trend is only clear for the g_m_ values at V_GS_ = +0.2 V with a variation of up to 30%, as achieved for the nanomolar concentration, in comparison to the initial value. This plot highlights the major sensitivity of the OECT response versus the antigen concentration in the intermediate V_GS_ region, in comparison with what is observed for lower values of V_GS_ (i.e., 0 and −0.2 V).

Moreover, in the graphs of Figure 5a,b (top panel), the I_DS_ modulation and transconductance at V_GS_ = +0.2 V values obtained for the experiments carried out with anti-NP protein functionalized gate electrode before and after incubation in a solution with 57 nM of spike-RBD protein are also included. In this case, because of the lack of a antigen–antibody complex, the I_DS_ modulation and g_m_ values before and after the incubation are completely indistinguishable.

With the aim of setting up an alternative and potentially more direct protocol to demonstrate the detection of the spike-RBD protein from the analysis of the OECT response, we elaborated the data through a different procedure focused on the modelling of the transfer curves region between −0.5 and 0.3 V. Hence, all the transfer curves in this V_GS_ range were modelled by a simple second-order polynomial (I_DS_$=\alpha +\beta \cdot V+\gamma \cdot V^{2})$. Figure S5 in the Supplementary File presents examples of this fitting procedure applied to the transfer curves initially introduced in Figure 3 and Figure 4a. The excellent quality of the fitting curves demonstrates the properness of the approach, which can model both the initial linear and the following quadratic dependence of I_DS_ on V_GS_.

**Figure 6:**
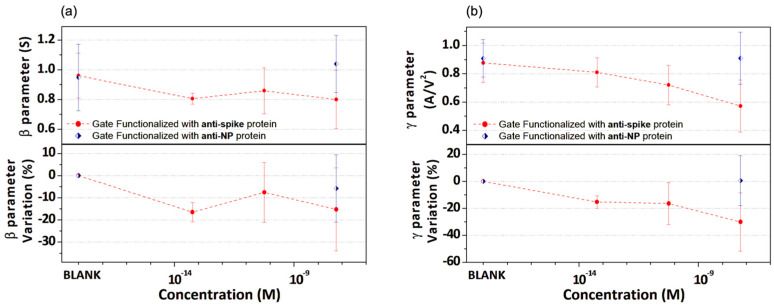
β and γ fitting parameters (top panels) and the corresponding percent variations (bottom panels) are reported in (**a**,**b**), respectively, as a function of the spike-RBD protein concentration for gate electrodes functionalized with spike and nucleocapside antibodies.

Hence, the values of the β and γ fitting parameters averaged for all the experiments performed at different spike-RBD protein concentrations are plotted in Figure 6a,b, respectively. In particular, while resembling the behavior of g_m_ at V_GS_ = 0 and −0.2 V, even if the β fitting parameter (Figure 6a) is significantly reduced in the lowest concentration (i.e., fM) range in comparison with the BLANK test (i.e., prior to any incubation step [36]), it does not exhibit a regular dependence on the spike-RBD protein concentration, particularly for the values in the pico- and nano-molar ranges. Conversely, the γ fitting parameter quite satisfactorily reproduces the behavior achieved for the g_m_ value estimated at the single V_GS_ = 0.2 V, displaying a monotonously decaying behavior as a function of the spike-RBD protein concentration for the anti-spike protein functionalized gate electrode. In this case, no significant variations were observed before and after incubation in a solution with 57 nM of spike-RBD protein solution when a gate functionalized with anti-NP protein was employed. Hence, this approach confirms that the V_GS_ region in which I_DS_ shows a square dependence on V_GS_ is the most sensitive to the changes produced by the gate incubation process. It should be outlined that the progressive occurrence of the g_m_ reduction in the OECT response, as a consequence of a protein detection at the gate/electrolyte interface, has been reported in other works, confirming that transconductance behavior is strictly related to the specific V_GS_ region [25]. This feature points to the need for alternative theoretical frameworks that can provide more insightful descriptions of the OECT electrical behavior [39,40].

In summary, in this work, we assessed the selective detection of spike-RBD proteins down to femto-molar range by using OECTs based on PEDOT:PSS channels. In addition to the overall I_DS_ modulation values, our analysis revealed that the electrical parameters associated to the transconductance (i.e., the slope of the I_DS_ *vs* V_GS_ transfer curves) and the V_GS_ region (between 0 and 0.3 V) of the OECT response are more clearly affected by the gate incubation process. The overall time for the testing procedure is about 30 min, with 20 min being required for the incubation step and the remaining time needed to perform the electrical analysis consisting of the recording of a number of consecutive transfer curves to be averaged. Given the discussed findings, at this stage, the sensitivity performances is estimated to be approximately in the range of few tens of nanomolars of the antigen concentration (corresponding to few hundreds of ng/mL of the spike-RBD protein). Further improvements can be achieved by optimizing the functionalization protocol or using alternative probes for antigen recognition [25]. Actually, as reported by a large number of works discussed in the literature, the sensitivity performances of devices conceived for the spike protein detection are strongly dependent (i.e., over several orders of magnitudes) on the specific sensing schemes [1,2]. It should be also mentioned that, in the analyzed configuration, the organic active channel immersed in PBS can be used for multiple experiments, in combination with different properly functionalized gate electrodes upon their incubation in the solution of interest. This approach simplifies the use of these devices which, owing to their low applied voltages, are suitable for further miniaturization and can be integrated in a compact and portable measurement set-up. Going beyond the precise diagnosis of coronavirus, all these features are very promising to the development of cheap, fast, and user-friendly tools for point-of-care analysis where OECT could be embedded in microfluidic–OECT-integrated platforms.

## Data Availability

Data are contained within the article or supplementary materials.

## Supplementary Materials

The following supporting information can be downloaded at: https://www.mdpi.com/article/10.3390/bios13070739/s1, Figure S1: FITR spectra recorded for a gold surface before (SAMPLE A) and after (SAMPLE B), showing the complete functionalization process with anti-spike antibody; Figure S2: I_GATE_ curves corresponding to the output (a) and transfer curves (b) reported, respectively, in Figure 2a,b of the main text; Figure S3: (a) Transfer curves achieved (V_DS_ = −0.3V) using different values of the time delay between the V_GS_ application and the I_DS_ recording, where the corresponding transconductance (g_m_) and I_GATE_ values are reported in (b) and (c), respectively; Figure S4: (a–c) Transconductance (g_m_) curves as a function of V_GS_ extracted from the transfer curves shown, respectively, in Figure 3a–c of the main text. (d) g_m_ curves evaluated from the transfer curves shown in Figure 4a; Figure S5: OECT transfer curves (scatter symbols) with V_DS_ = −0.3 V and related fitting curves with the equation I_DS_$=\left(\alpha +\beta \cdot V+\gamma \cdot V^{2}\right)$. (solid lines) achieved using a functionalized gate with anti-spike protein before incubation and after incubation in solutions with (a) 57 femtomolar (fM); (b) 57 picomolar (pM) and (c) 57 nanomolar (nM) spike-RBD protein; (d) Corresponding transfer curves and fitting lines obtanied for a functionalized gate with nucleocapside (NP) antibody before incubation and after incubation with 57 nanomolar spike-RBD protein solution.
