# Supplementary material for: Organic Electrochemical Transistor Immuno-Sensors for Spike Protein Early Detection
Source: Biosensors (Basel). 2023 Jul 17;13(7):739. doi: 10.3390/bios13070739 (PMC10377135; doi:10.3390/bios13070739)
Supplement: Supplementary file 1 [file biosensors-13-00739-s001.zip › biosensors-2480489-supplementary.pdf]

biosensors

MDPI

Supplementary

Organic Electrochemical Transistor Immuno-Sensors for Spike Protein Early Detection

Mario Barra [1,*], Giovanna Tomaiuolo [2,3], Valeria Rachela Villella [2,3], Speranza Esposito [2,3], Aris Liboà [4,5], Pasquale D'Angelo [4], Simone Luigi Marasso [4,6], Matteo Cocuzza [4,6], Valentina Bertana [6], Elena Camilli [6] and Valentina Preziosi [2,3,*]

1 CNR-SPIN, c/o Department of Physics ''Ettore Pancini'', P.le Tecchio, 80, 80125 Napoli, Italy
2 Department of Chemical, Materials and Production Engineering —University Federico II, P.le Tecchio 80, 80125 Napoli, Italy; g.tomaiuolo@unina.it (G.T.); valeria.villella@gmail.com (V.R.V.); speranza.esposito@gmail.com (S.E.)
3 CEINGE, Advanced Biotechnologies, 80145 Napoli, Italy
4 IMEM-CNR, Parco Area delle Scienze 37/A, 43124 Parma, Italy; aris.liboa@unipr.it (A.L.); pasquale.dangelo@imem.cnr.it (P.D.)
5 Graduate School in Science and Technologies of Materials and Department of Physics, University of Parma, Parco Area delle Scienze, 7/A, 43121 Parma, Italy
6 ChiLab, Department of Applied Science and Technology, Politecnico di Torino, 10129 Torino, Italy; simone.marasso@polito.it (S.L.M.); matteo.cocuzza@polito.it (M.C.); valentina.bertana@polito.it (V.B.); elena.camilli@studenti.polito.it (E.C.)
* Correspondence: mario.barra@spin.cnr.it (M.B.); valentina.preziosi@unina.it (V.P.); Tel.: +39-0817682428 (M.B.); +39-0817682539 (V.P.)

Citation: Barra, M.; Tomaiuolo, G.; Villella, V.R.; Esposito, S.; Liboà, A.; D'Angelo, P.; Marasso, S.L.; Cocuzza, M.; Bertana, V.; Camilli, E.; et al. Organic Electrochemical Transistor Immuno-Sensors for Spike Protein Early Detection. *Biosensors* **2023**, *12*, x. https://doi.org/10.3390/xxxxx

Received: 15 June 2023
Revised: 3 July 2023
Accepted: 5 July 2023
Published: 17 July 2023

Copyright: © 2023 by the authors. Submitted for possible open access publication under the terms and conditions of the Creative Commons Attribution (CC BY) license (https://creativecommons.org/licenses/by/4.0/).

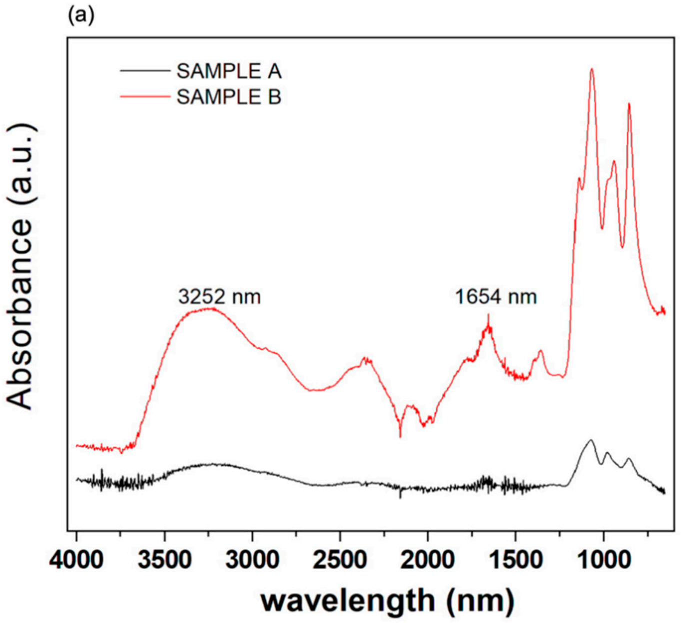

Figure S1. FITR spectra recorded for a gold surface before (SAMPLE A) and after (SAMPLE B) the complete functionalization process with anti-spike antibody.

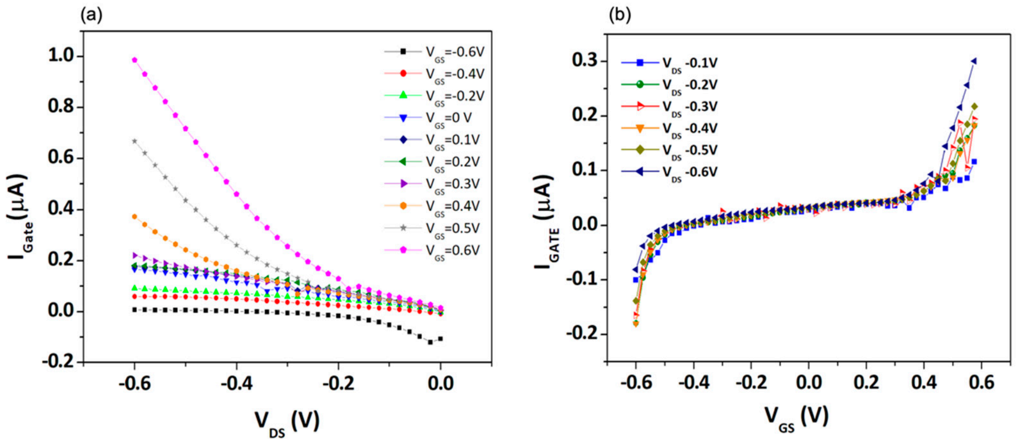

Figure S2. I_{GATE} curves corresponding to the output (**a**) and transfer curves (**b**) reported, respectively, in Fig.2a and 2b of the main text.

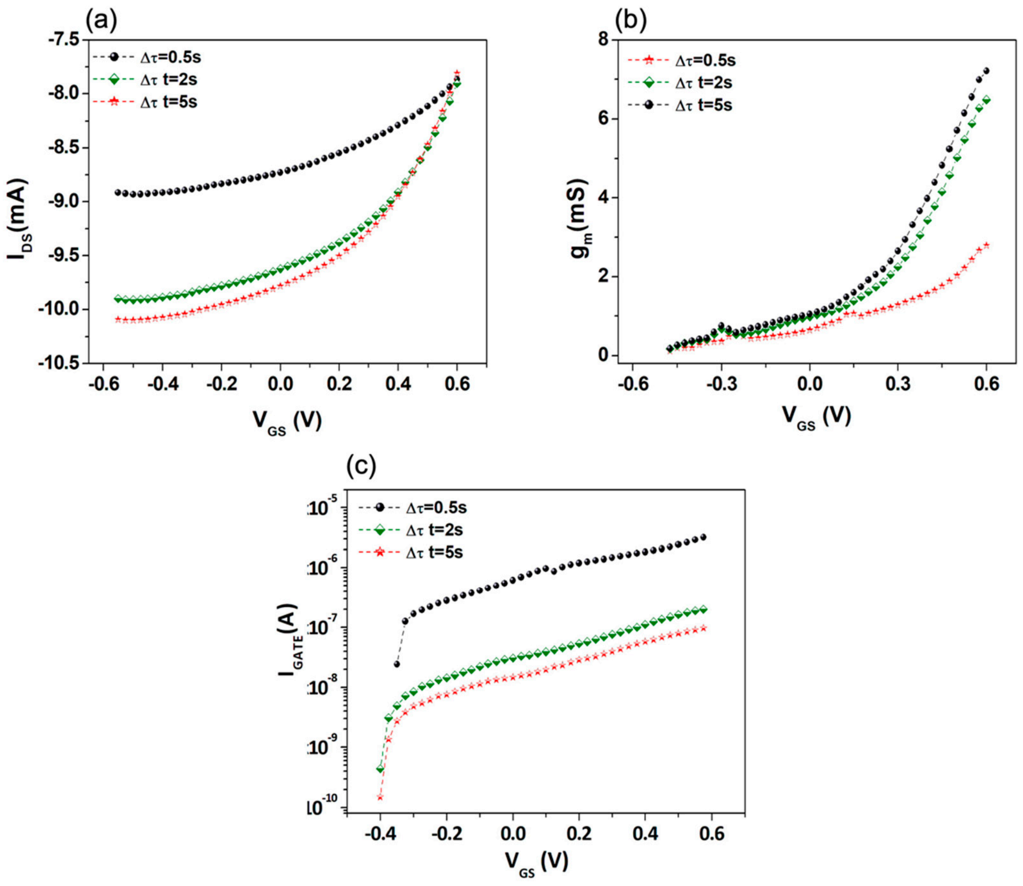

Figure S3. (**a**) Transfer curves achieved (V_{DS} = −0.3 V) using different values of the time delay between the V_{GS} application and the I_{DS} recording. The corresponding transconductance (g_m) and I_{GATE} values are reported in (**b**,**c**), respectively.

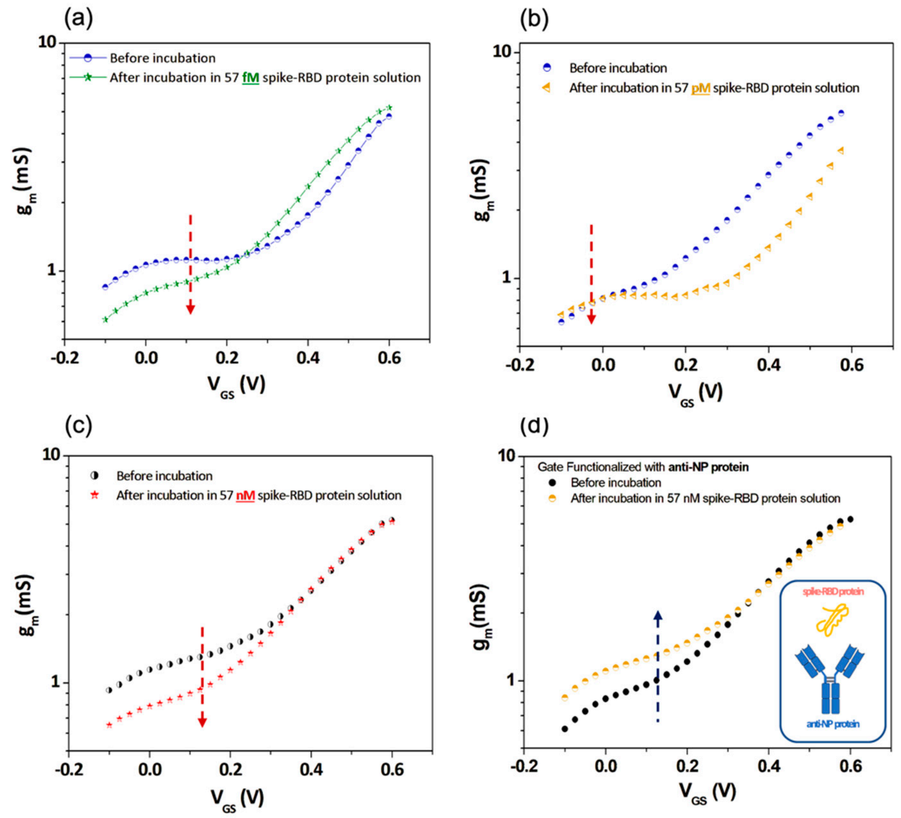

Figure S4. (**a**–**c**) Transconductance (g_m) curves as a function of V_{GS} extracted from the transfer-curves shown, respectively, in (**a**–**c**) of the main text; (**d**) g_m curves evaluated from the transfer curves shown in (**a**).

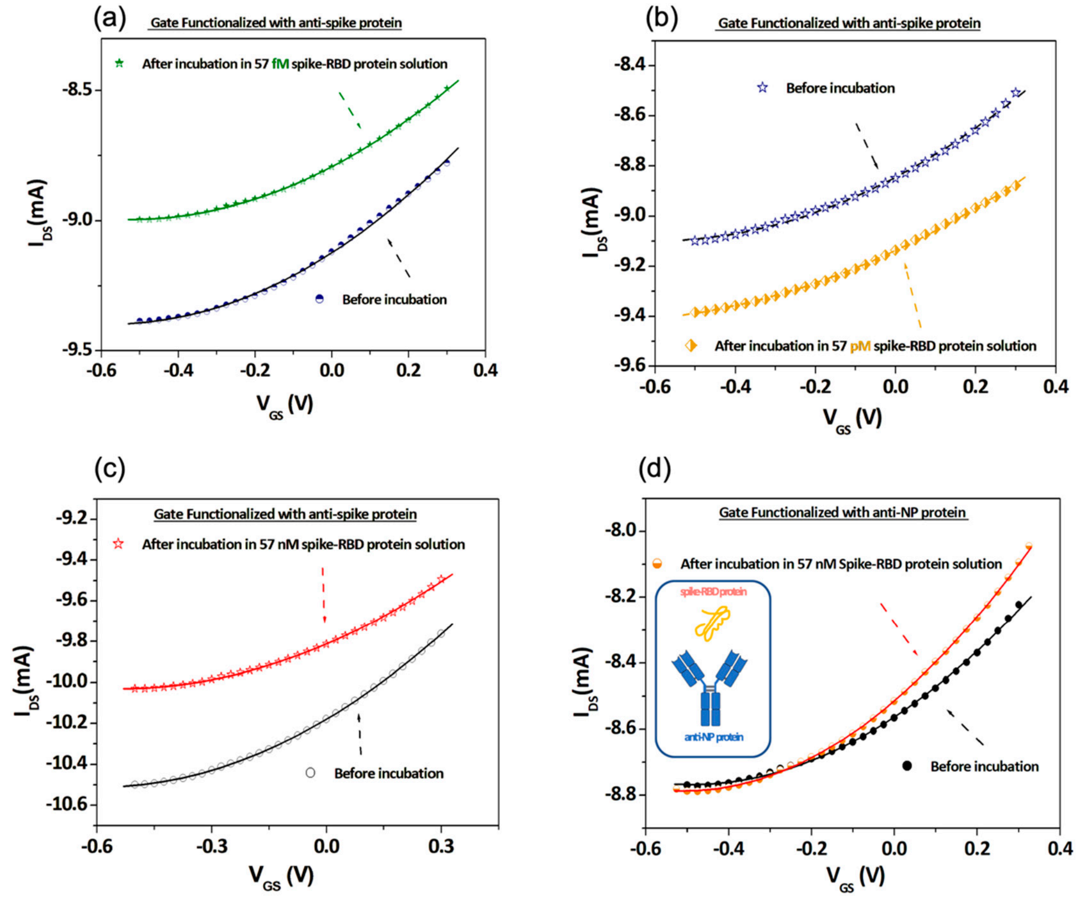

Figure S5. OECT transfer-curves (scatter symbols) with V_{DS} = −0.3 V and related fitting curves with the equation $I_{DS} = (\alpha + \beta \cdot V + \gamma \cdot V^2)$ (solid lines) achieved by using a functionalized gate with anti-spike protein before incubation and after incubation in solutions with (**a**) 57 femtomolar (fM); (**b**) 57 picomolar (pM) and (**c**) 57 nanomolar (nM) spike-RBD protein; (**d**) Corresponding transfer-curves and fitting lines obtanied for a functionalized gate with nucleocapside (NP) antibody before incubation and after incubation with 57 nanomolar spike-RBD protein solution.
